# COMT Val158Met polymorphism, cognitive stability and cognitive flexibility: an experimental examination

**DOI:** 10.1186/1744-9081-6-53

**Published:** 2010-09-13

**Authors:** Elise C Rosa, Dwight Dickinson, José Apud, Daniel R Weinberger, Brita Elvevåg

**Affiliations:** 1 Clinical Brain Disorders Branch, National Institute of Mental Heath/National Institutes of Health, 10 Center Drive, MSC 1379, Bethesda, MD 20892, USA

## Abstract

**Background:**

Dopamine in prefrontal cortex (PFC) modulates core cognitive processes, notably working memory and executive control. Dopamine regulating genes and polymorphisms affecting PFC - including Catechol-O-Methyltransferase (COMT) Val158Met - are crucial to understanding the molecular genetics of cognitive function and dysfunction. A mechanistic account of the COMT Val158Met effect associates the Met allele with increased tonic dopamine transmission underlying maintenance of relevant information, and the Val allele with increased phasic dopamine transmission underlying the flexibility of updating new information. Thus, consistent with some earlier work, we predicted that Val carriers would display poorer performance when the maintenance component was taxed, while Met carriers would be less efficient when rapid updating was required.

**Methods:**

Using a Stroop task that manipulated level of required cognitive stability and flexibility, we examined reaction time performance of patients with schizophrenia (n = 67) and healthy controls (n = 186) genotyped for the Val/Met variation.

**Results:**

In both groups we found a Met advantage for tasks requiring cognitive stability, but no COMT effect when a moderate level of cognitive flexibility was required, or when a conflict cost measure was calculated.

**Conclusions:**

Our results do not support a simple stability/flexibility model of dopamine COMT Val/Met effects and suggest a somewhat different conceptualization and experimental operationalization of these cognitive components.

## Background

Dopamine level in prefrontal cortex (PFC) and striatum modulates core cognitive processes by tuning neuronal and circuit responses [1,2]. Understanding genes that regulate dopamine in PFC - such as catechol-O-methyltransferase (COMT) gene on chromosome 22q11 - may help to unravel the complex neurobiological processes that underlie cognitive function and dysfunction in health and illness. A COMT polymorphism, Val158Met, plays a central role in cortical dopamine degradation, with the Val allele associated with greater COMT enzyme activity, greater dopamine degradation and less synaptic dopamine than the Met allele [3,4]. Val158Met has been linked to prefrontally mediated cognition (e.g., executive function and working memory - [5-7] - and attentional control - [8,9]; but see [10]). In particular, considerable evidence suggests that Met alleles are associated with more efficient patterns of prefrontal cortical activation and superior cognitive performance. This pattern has been observed in schizophrenia patients [5], and in most of the literature, COMT effects are independent of psychiatric diagnosis or risk status, suggesting that genotype modulates typical as well as impaired prefrontal cognition [5]. Given the complexity of clinical diagnosis, cognitive performance has been proposed as an intermediate phenotype for investigation of schizophrenia [11].

Much early work linking genes to neurobiology has relied on simple biological models and traditional neuropsychological measures (e.g., the Wisconsin Card Sorting Test). More recent investigations have proposed more detailed models and sophisticated psychometric assays to parse and segregate underlying cognitive processes, or at least restrict the processes by which one succeeds or fails on a task. For example, one biologically plausible account of the effects of Val158Met on cognition builds on emerging ideas about the roles of tonic and phasic dopamine in cognitive processing. This theory proposes that tonic stimulation of cortical D1 receptors stabilizes and maintains relevant information, while phasic stimulation of striatal D2 receptors underlies "cognitive flexibility", such as updating and manipulating information [2,12]. Extending the theory to Val158Met, it has been proposed that the Met allele is associated with increased tonic dopamine transmission, while the Val allele is associated with increased phasic dopamine transmission. Thus Met carriers would be expected to display greater efficiency and better performance on the elements of working memory and executive tasks that require stable maintenance of information, and Val carriers would be more efficient when cognitive flexibility is required, such as during rapid updating or task switching [13].

To test this hypothesis, one group of investigators used a response shifting paradigm, the Competing Programs Task, in a small sample of patients with schizophrenia and schizoaffective disorder (n = 26) [14]. The task required responding to an imitation rule (e.g., two cues followed by two button presses) during blocks 1 and 3 and a reversal rule (one cue followed by two button presses or two cues followed by one button press) during blocks 2 and 4. Participants transitioned between conditions and the rules changed after either a maximum of 20 trials, or after 8 consecutive correct responses were produced by the participant. Met homozygotes (n = 8) generated significantly faster responses than Val homozygotes (n = 6), were significantly more accurate in the imitation condition, and required significantly fewer trials-to-criterion for the imitation blocks. The authors interpreted these findings as support for an association between greater cognitive stability and the Met allele. Participants with Val alleles performed better on a derived measure intended to index cognitive flexibility [14].

Although the investigators reported their findings as consistent with the tonic/phasic dopamine hypothesis, this interpretation is not clear cut, for several reasons. As an initial matter, the Competing Programs Task may be better understood as a paradigm requiring different levels of cognitive control or conflict processing than as a task alternately requiring stable and flexible cognitive processing. Essentially, the imitation condition requires participants to emit a relatively intuitive response, suggested by the stimuli, while the reversal condition requires that participants resist the intuitive response and produce an exactly counter-intuitive response. In this regard the task presented in [14] is reminiscent of the classic Stroop paradigm, in which emphasis is first placed on relatively automatic word reading, and then on resisting automatic word reading in order to name incongruent colors in later trials. If the Competing Programs Task is better interpreted as a cognitive control or conflict processing paradigm, requiring focused response inhibition rather than flexible, fluid updating, it maps quite differently onto the tonic/phasic dopamine hypothesis. Recently, another study addressed this hypothesis through an analysis of the Attention Network Task (a version of the Flanker paradigm)[15]. This report refers specifically to the analysis in [14], but here the investigators framed this question as whether Val carriers might have an advantage relative to Met carriers in conflict processing rather than cognitive flexibility.

Importantly, in these studies, the putative Val advantage is open to question for parallel reasons. Although the paradigms (Competing Programs vs. Attention Network Task) and parameters (accuracy vs RT) are different, both studies calculate a "switching cost" that relates performance on the simpler congruent condition to performance on the more difficult conflict condition. In principle, the "Val advantage" on these cost indexes could be entirely accounted for by the loss of a significant Met advantage in the simpler processing condition, rather than any frank Val advantage. For example, [14] reported a Met allele trials-to-criterion advantage for the simpler, imitation blocks of their task, but no Met or Val allele advantage for the switching blocks, indicating the loss of a Met advantage, but not the emergence of a Val advantage.

A further point is that the broad literature addressing the relationship of Val158Met with cognition is difficult to reconcile with a sharp genotype-based distinction between stable and flexible cognitive processing. Generally, this literature shows a Met advantage across various tasks that seem to mix demands for stable and flexible cognitive processing. For example, several reports have documented a Met advantage on a spatial working memory task that requires both maintenance of information and also continuous updating of the contents of working memory (e.g., [16]), on a numerical computation task that required performing subtraction then making a size comparison [17], and, in children, on a dots-mixed task requiring working memory and inhibition [18]). Additionally, the Met allele has been specifically associated with speeded low-level lexical access (on a letter comparison task, but not on simple pattern comparison [19]), and with a speed advantage for response times [20]. Finally, we are not aware of consistent findings of Val allele advantages in any reported cognitive tasks.

To help clarify these issues, we conducted an analysis of Val158Met effects roughly parallel to [14], using the more familiar Stroop paradigm, in a large sample of both patients with schizophrenia and healthy controls. We (i) challenged participants by utilizing a cognitive assay that manipulated the level of required cognitive control within the same task, and (ii) examined whether Met carriers would display a performance advantage only when the cognitive control requirement was low, and Val carriers would perform better when the demand for cognitive control was increased.

## Methods

### Participants

Sixty-seven patients with a DSM-IV diagnosis of schizophrenia or schizoaffective disorder and 168 healthy volunteers participated (see Table 1). Our protocol was approved by an Institutional Review Board, and all participants gave written informed consent, in accordance with the Helsinki Declaration.

**Table 1:** Age, gender, IQ and COMT genotype distribution of patients and controls

Variable	Patients (*n *= 67)	Controls(*n *= 186)
Age (years) - mean, SD	34, 9.89	36, 10.88
Females/Males	14/53	95/91
WAIS-R IQ *- mean, range	93, 70-113	108, 87-129
WRAT-R IQ* - mean, range	103, 73-120	109, 81-127
Val/Val, Val/Met, Met/Met	17, 31, 19	43, 97, 46

*p < .05

### Design and procedure

As part of the NIMH Sibling Study [5], all participants completed a neuropsychological test battery to determine cognitive functioning. Additionally, a behavioral Stroop task was presented via a computer monitor that required rapid and accurate responding by pressing buttons labeled "red" (right button) or "green" (left button) to the words "green" and "red" that appeared one at a time. The level of task demands was manipulated by presenting either congruent or incongruent test items (e.g., "green" written in green ink, versus "green" written in red ink), and by either keeping the cue - and thus task - demands constant during portions of the task (i.e., responding to the word or responding to ink color) or switching the cue randomly in other portions, and thus introducing frequently shifting task demands.

Stimuli remained on the screen until a button was pushed, or for a maximum of 3000 msecs. Median reaction times (RTs) for correct trials were computed to control for outliers. Reaction time data was used for this analysis, as even in the most difficult switching condition participants were fairly accurate: patients' average = 89.75%, healthy volunteers' average = 94.04%. COMT Val158Met (rs4680) genotyping was performed on DNA obtained from B lymphoblast cell lines using the Taqman 5'-exonuclease assay as described previously [3]. There were no significant genotype differences by age, gender, or WRAT-IQ in patients or healthy volunteers (All *F*'s < 2.13, all *p*'s > 0.13).

## Results

First, given the large element of automaticity in reading, and the simple but rapid mapping required between lexical-semantic aspects of the stimuli and response, we predicted the Met allele would be associated with superior performance when the demand for cognitive flexibility and/or control was low, namely simple reading of the words "green" and "red" (in black ink).

**Figure 1:**
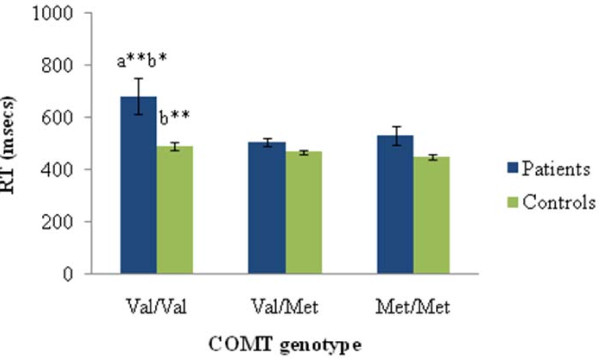
**Average reaction times (msecs) of reading and responding to the words "red" and "green" written in black ink in patients and controls as a function of COMT genotype (24 trials)**. Error bars represent standard error. a = vs Val/Met. b = vs Met/Met. *p < .055. **p < .01

As expected, patients' performance was overall slower than controls (*F*(1,251) = 27.93, *p *< .0001), but qualitatively similar: In patients, there was a Met allele RT advantage (*F*(2,64) = 5.87, *p *= .004; effect size (Cohen's *f*) = .38 [21]) (see Figure 1). This Met advantage was also associated with general speed in participants with schizophrenia (*F*(2,63) = 4.77, *p *= 0.01; effect size (Cohen's *f*) = .33) on a composite of speeded neuropsychological tasks (Trails-A, Trails-B, and Digit-Symbol task from the WAIS-R, see [22] for details of composite speed measure), and is consistent in magnitude with a previous report of a Met allele advantage in patients with schizophrenia on these three neuropsychological tasks [23]. In our controls there was a Met allele RT advantage (*F*(2,183) = 3.00, *p *= .05; effect size (Cohen's *f*) = .14) but this was not related to the general speed composite. Thus, as predicted, there was a Met advantage on the speed of response during a relatively undemanding facet of this task.

**Figure 2:**
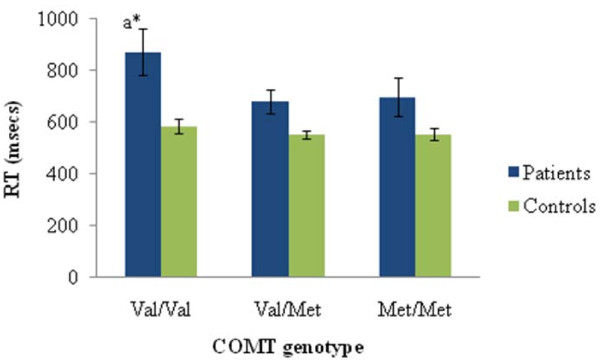
**Average reaction times (msecs) in patients and controls as a function of COMT genotype of naming the ink color (red or green) of the alternate word (red or green) (12 trials total)**. Error bars represent standard error. a = vs Val/Met. *p < .05

Second, we investigated whether the Met allele advantage would disappear once the task demands included a high control/flexibility component, as found with the reversal condition in [14]. Within our reversal condition, which required responding to the color (red or green) of the opposite word (red or green), there was no effect of genotype on reaction time among patients (*F*(2,64) = 2.31, *p *= 0.11)(although the Val/Val patient group's average reaction time was slower than the Val/Met's, consistent with an overall Met advantage), or controls (*F*(2,183) = 0.7, *p *= 0.50) (see Figure 2).

**Figure 3:**
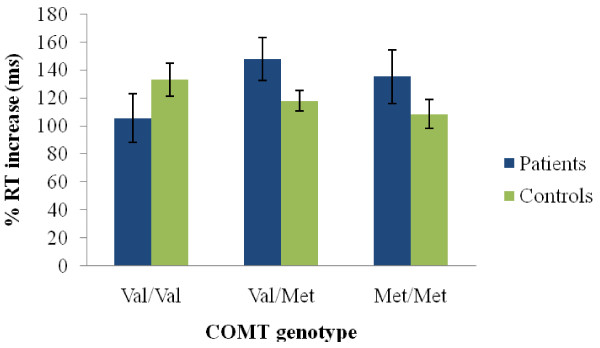
**Average reaction times (msecs) in patients and controls as a function of COMT genotype of responding to the cue to name the ink color of the word minus the average reaction times of responding to the ink colors of neutral symbols, divided by the reaction times of responding to the neutral symbols (6 word trials, 24 symbol trials)**. Error bars represent standard error. Post-hoc t-tests revealed no significant differences between COMT groups either within patients or within controls (all *p*'s > .05).

Third, we investigated whether our derived measure of "conflict cost"- calculated as the percent increase in reaction time between the Neutral Color trials and the cued Incongruent Color trials - would reveal a Val allele advantage, analogously to the report of [14]. We found no effect of genotype on this measure among our patients (*F*(2,64) = 1.48, *p *= 0.23) or controls (*F*(2,183) = 1.22, *p *= 0.29) (see Figure 3).

## Discussion

We applied COMT Val158Met genotype to a familiar cognitive assay in order to re-examine the explanatory power of the tonic/phasic dopamine hypothesis for cognitive stability and flexibility, task characteristics that are inherent - and likely confounded - in many measures of working memory and executive function. We attempted to replicate the findings of [14], that Met carriers display a performance advantage when the simpler maintenance component is taxed, while Val carriers are more efficient when more flexible processing is required. Our results were inconsistent with this hypothesis. Although our experimental operationalization of these cognitive components contributes to the growing literature on the superiority of Met alleles in working memory tasks, we did not uncover any frank advantage of the Val alleles in any condition. Our analyses of 'switching costs', the parameter that has been used to operationalize 'flexibility' in earlier examinations of this hypothesis, were non-significant. Additionally, this pattern, although non-significant, was in the direction we would expect if all that were occurring was a loss of the Met advantage. Specifically, it appears that the Met/Mets and Val/Mets have higher 'costs' on this task, without any evidence of a Val/Val advantage, and this result is similar (as far as we can tell) to that of earlier reports.

Furthermore, we would argue that previous operationalizations of the hypothesis have strayed from its initial conception insofar as they have relied on versions of the 'switching cost' parameter. As discussed earlier, whether in the context of the Competing Programs Task (14), the Attention Network Test (15), or the Stroop paradigm analyzed here, these parameters measure effortful cognitive control or conflict processing rather than the fluid, flexible cognitive processing that appears to be anticipated by the tonic/phasic dopamine model, and therefore do not squarely address the effects of dopamine activity on flexibility. Even with these differing interpretations, this study and most others have failed to find a consistent Val advantage for any tasks or conditions. This study adds to the literature of a Met advantage for tasks demanding cognitive stability, and acknowledges the need for a more refined model of the relationship between dopamine physiology and cognition.

Although the Tonic/Phasic Dopamine hypothesis yields an elegant model, a convincing demonstration of a Val advantage has yet to be reported. Nolan et al. attempted to capture the basics of the model, however, the task examined in that study likely confounded cognitive control with cognitive flexibility. Even with a similar operationalization we failed to replicate the findings of [14], and our results are more consistent with the broader literature indicating a general Met allele advantage and no frank Val advantages. Beyond psychometric differences, this story is likely to be magnitudes more complex with haplotype, gene-gene interaction, and gene-environment interaction effects on this behavioral phenotype. Indeed, our study underscores how much remains to be discovered concerning the role of functional polymorphisms in cognitive function and the need for further research in order to fine-tune useful clinical intermediate phenotypes that affect high-level information processing.

### Limitations

Although relatively large for such a study, it would be beneficial in the future to employ a bigger sample so that the role of other variables (e.g., gender) can be examined. This would afford sufficient power to undertake a meaningful and sophisticated analysis of the effect of COMT Val/Met on trial-by-trial performance so as to determine switching costs between individual trials, specifically between congruent and incongruent trials.

## Conclusions

Our data do not support a simple tonic/phasic model of COMT Val/Met effects. We found a Met allele advantage for tasks requiring cognitive stability, but no frank Val advantage for tasks of cognitive stability or cognitive flexibility.

## Competing interests

The authors declare that they have no competing interests.

## Authors' contributions

ECR tested participants, analyzed data and drafted the manuscript. DD provided assistance with data interpretation and manuscript revision. JA and DRW provided assistance with manuscript revision. BE designed the study, and helped with the analysis, interpretation, and writing of the manuscript. All authors read and approved the final manuscript.
